# Metabolic regulation analysis of an ethanologenic *Escherichia coli *strain based on RT-PCR and enzymatic activities

**DOI:** 10.1186/1754-6834-1-8

**Published:** 2008-05-01

**Authors:** Montserrat Orencio-Trejo, Noemí Flores, Adelfo Escalante, Georgina Hernández-Chávez, Francisco Bolívar, Guillermo Gosset, Alfredo Martinez

**Affiliations:** 1 Departamento de Ingeniería Celular y Biocatálisis, Instituto de Biotecnología, Universidad Nacional Autónoma de México. Cuernavaca, Mor., México

## Abstract

**Background:**

A metabolic regulation study was performed, based upon measurements of enzymatic activities, fermentation performance, and RT-PCR analysis of pathways related to central carbon metabolism, in an ethanologenic *Escherichia coli *strain (CCE14) derived from lineage C. In comparison with previous engineered strains, this *E coli *derivative has a higher ethanol production rate in mineral medium, as a result of the elevated heterologous expression of the chromosomally integrated genes encoding PDC_*Zm *_and ADH_*Zm *_(pyruvate decarboxylase and alcohol dehydrogenase from *Zymomonas mobilis*). It is suggested that this behavior might be due to lineage differences between *E. coli *W and C.

**Results:**

This study demonstrated that the glycolytic flux is controlled, in this case, by reactions outside glycolysis, *i.e.*, the fermentative pathways. Changes in ethanol production rate in this ethanologenic strain result in low organic acid production rates, and high glycolytic and ethanologenic fluxes, that correlate with enhanced transcription and enzymatic activity levels of PDC_*Zm *_and ADH_*Zm*_. Furthermore, a higher ethanol yield (90% of the theoretical) in glucose-mineral media was obtained with CCE14 in comparison with previous engineered *E. coli *strains, such as KO11, that produces a 70% yield under the same conditions.

**Conclusion:**

Results suggest that a higher ethanol formation rate, caused by ahigher PDC_*Zm *_and ADH_*Zm *_activities induces a metabolic state that cells compensate through enhanced glucose transport, ATP synthesis, and NAD-NADH+H turnover rates. These results show that glycolytic enzymatic activities, present in *E. coli *W and C under fermentative conditions, are sufficient to contend with increases in glucose consumption and product formation rates.

## Background

Fermentative metabolism constitutes a fundamental cellular capacity for industrial biocatalysis. Endogenous organic compounds used by cells as terminal electron acceptors under oxygen deprivation are converted into biochemical products that are waste products for the cell, such as ethanol, lactate, acetate, succinate, formate and hydrogen, but represent valuable molecules to society [1]. For example, renewable fuels from biomass, such as ethanol, constitute energy sources that preserve the environment since the carbon dioxide released from their combustion can be integrated into a photosynthetic cycle, which does not participate in a net carbon dioxide buildup into the atmosphere.

Metabolic engineering strategies have been used to modify microorganisms to convert all sugars arising from chemical-enzymatic hydrolysis of lignocellulose, such as xylose, arabinose, and glucose into ethanol. A wide variety of research approaches have been employed for this purpose; among the most effective attempts are the engineering of different Gram-negative bacteria, such as *Escherichia coli *[2-6], *Klebsiella oxytoca *[7-9] and *Zymomonas mobilis *[10,11] as well as yeast, such as *Saccharomyces cerevisiae *[12-16]. One of the most successful strategies to develop ethanologenic bacteria was developed by Ingram and co-workers [2,3,6-8,17]. In the case of *E. coli*, the W strain was engineered for ethanol production by integrating the pyruvate decarboxylase (*pdc*) and alcohol dehydrogenase (*adhII*) genes from *Z. mobilis*, under the control of the *pflB *promoter, to obtain strain KO11 [3,17]. Expression from this promoter is high under anaerobic conditions [18,19], and ethanologenic *E. coli *strains, such as KO11 and LY01 have shown to be efficient in the conversion of all sugars present in lignocellulosic hydrolysates into ethanol [20,21].

Expression profiling is a powerful tool for analyzing gene transcription at a genomic scale. It can be used to compare global relative changes in gene expression that occur in response to an environmental stimulus or to compare the effects of genetic modifications on gene expression. This type of analysis can provide important information about cell physiology and has the potential to identify connections between regulatory or metabolic pathways not previously known [22,23]. Since the physiological state and fermentation performance of a cell is dictated primarily at the protein level, transcription results should be complemented by determining specific enzyme activities to provide a better understanding of the observed phenomenon, considering that enzymatic and transcriptional regulation mechanisms are different [22].

Previous studies have shown that plasmid-encoded levels of *Z. mobilis *pyruvate decarboxylase (PDC_*Zm*_) and alcohol dehydrogenase II (ADH_*Zm*_) in *E. coli *correlate with the titer and the formation rate of ethanol [24,21,25]. Furthermore, the introduction of this heterologous pathway has several effects on *E. coli *physiology under fermentative conditions, *i.e*., increases its growth rate and glycolytic flux when cultivated in Luria Broth with xylose [26] or glucose [27]. Gene array studies have also shown that several genes from the pentose phosphate and glycolytic pathways have statistically significant higher expression levels when ethanologenic *E. coli *(strain KO11) ferments xylose [26,27].

The present study was conducted to understand the role that chromosomally integrated *pdc*_*Zm *_and *adh*_*Zm *_heterologous expression has on the physiology and metabolic performance of *E. coli *during glucose fermentation in mineral media. The regulation of metabolic pathways, related to central carbon metabolism and fermentation performance, was studied using mainly the measurements for both the enzymatic activities of the glycolytic and fermentative pathways, as well as transcript levels from genes coding for the enzymes involved in the glycolytic, pentose phosphate, and fermentative pathways. Glucose transporters and anaerobic regulators were also analyzed using transcriptome data. Evaluation was performed using wild type *E. coli *C as the reference strain, and a new ethanologenic strain derived from *E. coli *C, CCE14 (*E. coli *C: *pflB::pdc adhB cat*). Interestingly, strain CCE14 has ca. five-fold higher values of PDC_*Zm *_and ADH_*Zm *_enzymatic activities than strain KO11 (*E. coli *W: *pflB::pdc adhB cat, Δfrd*) [3,17]. The results show that not only the specific ethanol rate, but also the glucose consumption rate (glycolytic flux) are increased as pyruvate decarboxylase and alcohol dehydrogenase transcripts and enzymatic activities are increased. Moreover, glycolytic flux is controlled by reactions outside glycolysis.

## Results and discussion

### Effect of PDC_Zm _and ADH_Zm _activity levels on fermentation performance

**Figure 1:**
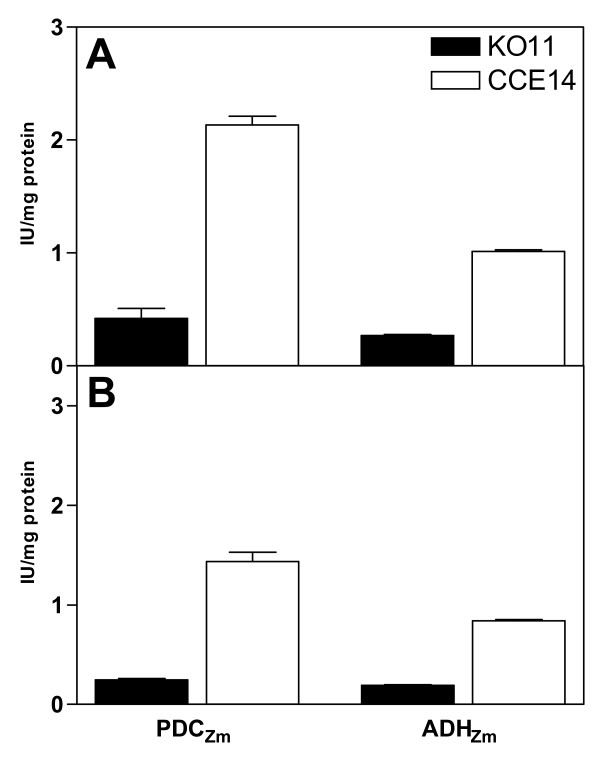
Specific enzyme activities values of PDC_Zm _and ADH_Zm _for strains KO11 and CCE14 during exponential (A) and stationary phases (B).

In comparison with the KO11 strain, PDC_*Zm *_and ADH_*Zm *_enzymatic specific activities were on average, 5 and 4-fold larger, respectively, in CCE14 during both exponential and stationary phases (Fig. 1). Even though the heterologous pathway was integrated into the chromosome of strains KO11 and CCE14 using the same method (see materials and methods section), strong differences were encountered between PDC_*Zm *_and ADH_*Zm *_enzymatic activities, as well as in transcript levels (as shown below). This behavior might be due to lineage differences between KO11 and CCE14. It is noteworthy that strain KO11 was submitted to high chloramphenicol pressure selection to increase ethanol productivity [3], in spite of this additional strategy, PDC_*Zm *_and ADH_*Zm *_activities were higher in CCE14, wich was not further selected on high chloramphenicol.

**Figure 2:**
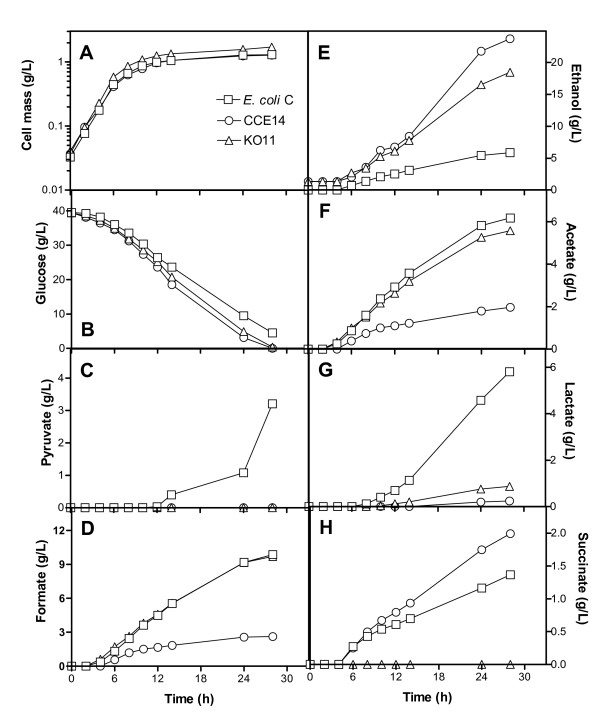
Characterization of *E. coli *C and ethanologenic strains CCE14 and KO11 in M9 mineral media with 40 g/l glucose. Cell mass formation (A), Glucose (B), Pyruvate (C), Formate (D), Ethanol (E), Acetate (F), Lactate (G), and Succinate (H).

Fermentation performance in mineral medium with 40 g/L glucose is presented in Fig. 2. Fig. 2A–B shows results obtained for cell mass formation and glucose consumption. Table 2 summarizes rates obtained during the exponential and stationary phases. The growth rates of *E. coli *C, CCE14, and KO11 were similar. During exponential growth, specific glucose consumption rates of the two ethanologenic strains (KO11 and CCE14) were 28 and 34% higher, respectively, than that obtained for *E. coli *C, indicating that the glycolytic flux increased as a result of *pdc*_*Zm *_and *adh*_*Zm *_expressions. This behavior correlates well with previously reported results for KO11 fermenting xylose [26] or glucose [27] in Luria Broth, where the maximum sugar consumption rate was 50% higher for KO11 than for strain W. In spite of the fact that the growth rate was similar for the three strains, the maximum cell mass obtained at the onset of the stationary phase (after 20 hours of fermentation) was similar for KO11 and *E. coli *C, and lower for CCE14 (Table 2). These results, and data presented in Fig. 1, indicate that CCE14 directs more carbon to ethanol production than to biomass biosynthesis.

**Table 2:** Kinetic constants in anaerobic cultures

Strain	μ	q_Glc _Exponential phase	q_Glc _Stationary phase	Cell Mass
*E. coli *C	0.42 ± 0.01	2.73 ± 0.02	1.11 ± 0.11	1.28 ± 0.07
CCE14	0.38 ± 0.01	3.68 ± 0.12	1.20 ± 0.10	1.07 ± 0.04
KO11	0.45 ± 0.01	3.49 ± 0.09	0.97 ± 0.06	1.39 ± 0.09

μ = Specific growth rate (1/h).

q_Glc _= Specific glucose consumption rate (g_Glc_/g_DCW _h).

Cell mass obtained at the onset of the stationary phase (g_DCW_/L).

± Standard deviation of three independent experiments.

Fig 2C shows that no pyruvate was secreted by ethanologenic strains, but *E. coli *C produced a significant amount (> 3 g/L) of this metabolite during the stationary phase (Fig 2C). Furthermore, formate production by KO11 and *E. coli *C were similar, reaching up to 9 g/L when glucose was exhausted (Fig. 2D). However, formate production was lower than 2 g/L for CCE14. PDC_*Zm *_was originally selected by Ohta et al [3], largely because it has a very high affinity for pyruvate (Km for pyruvate 0.4 mM) [28,29] in comparison with all competing fermentation enzymes [24]. This fact and our results indicate that competition occurs at the pyruvate node level, and that higher levels of PDC_*Zm *_and ADH_*Zm *_apparently allow more efficient carbon channeling through the heterologous ethanol pathway.

Although glycolytic fluxes for CCE14 and KO11 were similar, the ethanol specific formation rate in the exponential phase (first 6 hours of fermentation elapsed time) was 21% higher for CCE14 as compared to KO11 (Table 3). During this phase, the ethanol formation rate in *E. coli *C was negligible (Fig. 2E), whereas formate (Fig. 2D) and acetate (Fig. 2F) were the main products. The increase in ethanol production rate was obtained at the expense of acid production (Table 3, Fig. 2). Pyruvate and lactate (Fig. 2C, 2G) were not produced during this phase in CCE14, whereas formate, acetate, (Fig. 2D, 2F), and succinate (Fig. 2H) production were significantly lower than for *E. coli *C and KO11. Balances for this phase indicate carbon recoveries very close to 100% for the three strains evaluated (Table 3). These results indicate that the glycolytic flux can be controlled by the efficient conversion of pyruvate into ethanol through the enzymatic activity levels of PDC_*Zm *_and ADH_*Zm *_in ethanologenic *E. coli*. It is hypothesized that other fermentative pathways that allow the efficient regeneration of NAD^+ ^could have the same effect.

**Table 3:** Specific formation rates (q_P_) for organic acids and ethanol during the exponential phase (g_PRODUCT_/g_DCW _h)

Strain	Acetate	Formate	Succinate	Ethanol	Carbon Recovery (%)
*E. coli *C	0.95 ± 0.08	1.21 ± 0.03	0.29 ± 0.03	0.58 ± 0.05	100.9 ± 0.41
CCE14	0.39 ± 0.04	0.67 ± 0.04	0.28 ± 0.04	2.08 ± 0.01	104.5 ± 0.22
KO11	0.79 ± 0.04	1.59 ± 0.06	ND	1.63 ± 0.01	98.7 ± 0.32

± Standard deviation of three independent experiments.

ND. Not Detected.

During the stationary phase, CCE14 produced ethanol 80% faster than KO11 (Table 4). Accordingly, specific enzyme activities of PDC_*Zm *_and ADH_*Zm *_were 6 and 4.7-fold higher for CCE14 (Fig. 1). Ethanol yields at 30 hours were 15, 70 and 90% of theoretical yield for *E. coli *C, KO11, and CCE14, respectively.

**Table 4:** Specific formation rates (q_P_) for organic acids and ethanol during the stationary phase (g_PRODUCT_/g_DCW _h).

Strain	Acetate	Formate	Succinate	Lactate	Pyruvate	Ethanol
*E. coli *C	0.14 ± 0.07	0.26 ± 0.06	0.04 ± 0.01	0.26 ± 0.04	0.25 ± 0.02	0.12 ± 0.06
CCE14	0.04 ± 0.08	0.05 ± 0.04	0.06 ± 0.01	0.01 ± 0.015	ND	0.68 ± 0.01
KO11	0.10 ± 0.01	0.19 ± 0.02	ND	0.03 ± 0.05	ND	0.37 ± 0.03

± Standard deviation of three independent experiments.

ND. Not Detected.

### Metabolic regulation at transcript level

Transcript levels of 49 genes from the CCE14 strain were analyzed in the exponential growth phase and normalized for values obtained with *E. coli *C. Levels of *pdc*_*Zm *_and *adh*_*Zm *_were normalized with KO11 values and analyzed for both exponential and stationary phases. A Student' *t*-test with a *p *value of ≤ 0.05 was applied to each set of normalized values in order to determine statistical significant differences in expression levels.

**Figure 3:**
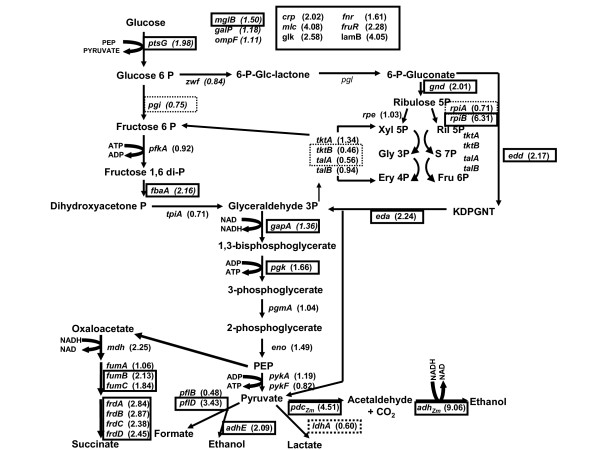
RT-PCR values for strain CCE14 normalized to *E. coli *C during the exponential phase. Higher values are represented in a continuous borderline and lower values are in dotted borderline. A *t*-student test with a *p *value of ≤ 0.05 was applied to each set of normalized values in order to determinate statistical differences in expression levels. Glucose transporter protein EIICB^Glc ^(*ptsG*), Glucokinase (*glk*), Phosphoglucose isomerase (*pgi*), Phosphofructokinase (*pfk*), Fructose bisphosphate aldolase (*fbaA*), Triose Phosphate isomerase (*tpi*), Glyceraldehyde-3P dehydrogenase (*gapA*), Phosphoglycerate kinase (*pgk*), Phosphoglycerate mutase (*pgmA*), Enolase (*eno*), Pyruvate kinase A (*pykA*), Pyruvate kinase B (*pykB*), Glucose-6P-1-dehydrogenase (*zwf*), 6-Phosphogluconate dehydrogenase (*gnd*), Ribulose phosphate epimerase (*rpe*), Ribose-5-phosphate isomerase A (*rpiA*), Ribose-5-phosphate isomerase B (*rpiB*), Transketolase A (*tktA*), Transketolase B (*tktB*), Transaldolase A (*talA*), Transaldolase B (*talB*), 2-keto-3-deoxy-phosphogluconate aldolase (*eda*), Phosphogluconate dehydratase (*edd*), Xylulose-5-phosphate (Xyl 5P), Ribulose-5-phosphate (Ril 5P), Glyceldehyde-3-phosphate (Gly 3P), Sedoheptulose-7-phosphate (S 7P), Erytrose 4-phosphate (Ery 4P), Fructose 6-phosphate (Fru 6P), 2-keto-3-deoxy-gluconate-6-phosphate (KDPGNT), Fumarate reductase A (*frdA*), Fumarate reductase B (*frdB*), Fumarate reductase C (*frdC*), Fumarate reductase D (*frdD*), pyruvate formate lyase B (*pflB*), pyruvate formate lyase D (*pflD*), Malate dehydrogenase (*mdh*), Fumarase A (*fumA*), Fumarase B (*fumB*), Fumarase C (*fumC*), alcohol dehydrogenase (*adhE*), lactate dehydrogenase (*ldhA*), Transcriptional regulator CRP (*crp*), Transcriptional repressor MLC (*mlc*), Transcriptional repressor MLC (*mlc*), Transcriptional regulator FRUR (*fruR*), High affinity maltose receptor (*lamB*), Galactose ABC transporter (*mglB*), Galactose permease (*galP*), *Zymomonas mobilis *alcohol dehydrogenase (*adh*_*Zm*_), *Zymomonas mobilis *pyruvate dehydrogenase (*pdc*_*Zm*_).

Interestingly, the comparison of CCE14 and KO11 gave a direct relationship between increases in both *pdc*_*Zm *_and *adh*_*Zm *_transcript values (4.51 and 9.06-fold, respectively) (Fig. 3) and specific enzyme activity levels (5.1 and 3.8-fold, respectively) (Fig. 1). It has been reported that *pdc*_*Zm *_and *adh*_*Zm *_mRNA are more stable than other transcripts in *Z. mobilis *[30]. A correlation was also found between CCE14 and KO11 during the stationary phase for transcripts (3.66-fold and 4.78-fold for *pdc*_*Zm *_and *adh*_*Zm*_, respectively) and specific enzyme activity levels (6-fold and 4.7-fold, respectively) (Fig. 1). Nevertheless, absolute values of the enzyme activity for these enzymes decreased around 50% during the stationary phase. The increase in specific ethanol formation rate (Table 4) correlated with higher transcript (Fig. 3) and specific enzyme activity (Fig. 1) levels for CCE14 in comparison to KO11. The requirements of higher PDC_*Zm *_enzymatic levels were demonstrated by Huerta-Beristain and co-workers [25]. These authors reported that when PDC_*Zm *_activity increased 7-fold, using a multicopy plasmid, the yield of ethanol from glucose increased from 70 to 85%, whereas organic acid formation rates were reduced in the KO11 strain. Accordingly, results in Fig. 1 clearly show that increases in the specific activities of the enzymes participating in the ethanologenic pathway boost the carbon flow to ethanol in strain CCE14. These results also suggest that high levels of both enzymes are essential to increase the ethanol production rate. Pyruvate pools in ethanologenic *E. coli *are substantially reduced by high-level expression of these *Z. mobilis *genes, but cells adjust their metabolism to various levels along the glycolytic pathway to fulfill the carbon flux. Direct increments between transcripts and specific enzyme activities demonstrate the correct translation of pyruvate decarboxylase and alcohol dehydrogenase when they are over expressed.

#### Glucose transport and phosphorilation

**Figure 4:**
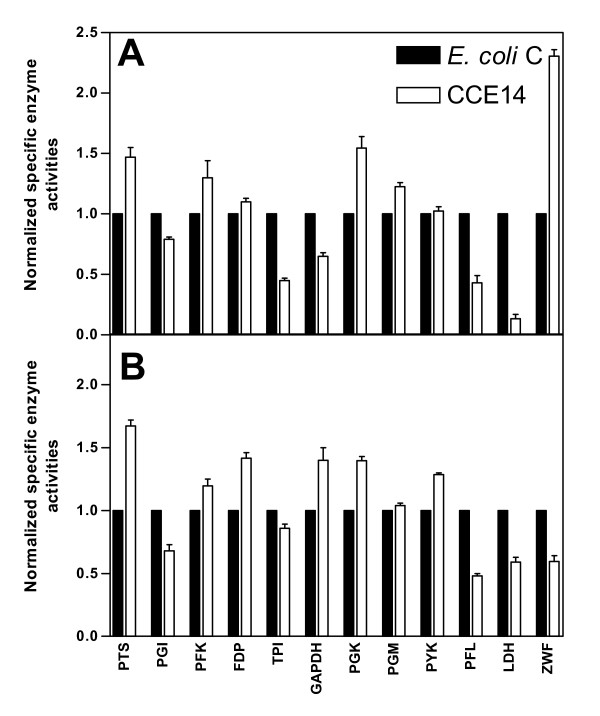
Specific enzyme activities for ethanologenic strain CCE14 normalized to parental strain *E. coli *C during exponential (A) and stationary phases (B) of culture. All measurements were performed in triplicate. The results presented are three independent experiments. A *t*-student test with a *p *value of ≤ 0.05 was applied to each set of normalized values in order to determinate statistical differences enzyme activity levels. Glucose – PEP phosphostransferase (PTS), glucose-6-phosphate dehydrogenase (ZWF), glucose phosphate isomerase (PGI), 6-phosphofructosekinase (PFK), fructose-1,6-bisphosphatase (FDP), fructose bisphospate aldolase (FDP aldolase), triose phosphate isomerase (TPI), glyceldehyde-3-phosphate dehydrogenase (GAPDH), 3-phosphoglycerate kinase (PGK), phosphoglycerate mutase (PGM), pyruvate kynase (PYK), pyruvate-formate lyase (PFL), lactate dehydrogenase (LDH), alcohol dehydrogenase (ADH_*Zm*_) and pyruvate decarboxylase (PDC_Zm_).

Genes coding for proteins related to transport and regulation of glucose such as *ptsG *(1.98-fold), transcriptional repressor coding *mlc *(4.08-fold) [31,32], high-affinity glucose transporters coding *lamB *(4.05-fold) and *mglB *(1.50-fold) [33], glucose kinase *glk *(2.58-fold), *fruR *(2.28-fold), and *crp *(2.02-fold) were more highly expressed in CCE14 than in *E. coli *C (Fig. 3). Specific glucose consumption rates (Table 2) and transport activities (Fig. 4) were on average 30 and 50% higher, respectively, for CCE14 in comparison with *E. coli *C. When glucose is phosphorylated in the course of transport, PTS proteins are dephosphorylated; unphosphorylated EIICB^Glc ^causes the formation of the Mlc-EIICB^Glc ^complex, derepressing the expression of target genes, such as *mlc *itself, as well as the *pts *operon [32]. Therefore, higher levels of *ptsG *and *mlc *correlate with increases in IICB^Glc ^(*ptsG *product) and MLC protein. The observed transcriptional pattern is consistent with a response that results in higher synthesis capacity for more PTS proteins necessary for glucose uptake.

On the other hand, wild type *E. coli *strains, growing on micromolar concentrations of glucose, synthetize galactose and maltodextrines as autoinducers derepressing the synthesis of the high-affinity glucose transport systems (MGLB and the LAMB maltoporin), which are responsible for glucose transport under these conditions [33]. Analyses of gene expression response in wild type *E. coli *from glucose non-limiting to glucose-limiting growth conditions, in chemostat cultures, have demonstrated that several genes including *mglB *and *lamB *are upregulated [34]. Furthermore, the *crp *transcript is higher when *E. coli *experience glucose limitation [35] and CRP regulates genes such as *mglB*, *lamb, glk*, and *ptsG *[36]. Our results show that even though cultures were growing in large amounts of glucose (40 g/L) throughout the exponential phase (Fig. 2), transcription results suggest that CCE14 was sensing partial glucose limitation. This response might be due to increases in the fermentation and glycolytic rates that in turn will induce a response to scavenge sugar through transport activation of alternative glucose transporters. Interestingly, *glk *transcript levels were significantly higher in CCE14. This result suggests that besides the PTS system, glucose could also be transported by MGLB and LAMB and phosphorylated by GLK. The higher *glk *transcript is also related to an elicited response due to the over expression of heterologous genes as demonstrated by Arora and Pederson [37].

As mentioned earlier, *fruR *(2.28-fold) was more highly expressed in CCE14. FRUR (or CRA) is a key regulator controlling the balance between glycolysis and gluconeogenesis [38-40]. Many gluconeogenic genes are activated by FRUR, while glycolytic genes such as *glk, pfkA, gapA, eno*, and *pykF *are repressed. [41,42]. However, none of the genes studied above was repressed. Probably FRUR was partially inactivated in the presence of glucose, because fructose-1-phosphate and fructose-1,6-bisphosphate bind to FRUR and inactivate its DNA-binding capacity [43,44].

#### Glycolytic pathway

In general, only slight changes were detected in the transcription level of genes related to glycolysis. For instance, *gapA *(1.36-fold), *fbaA *(2.16-fold), and *pgk *(1.66-fold) transcript levels were higher in CCE14 strain than in *E. coli *C (Fig. 3). In *E. coli*, the *gapA *gene is transcribed from at least four promoters, three recognized by the RNA polymerase Eσ^70 ^and one by the heat shock RNA polymerase Eσ^32^. This complex region of differentially regulated promoters allows the production of large amounts of *gapA *transcripts in a wide variety of environmental conditions [45]. On the other hand, in γ-proteobacteria (*E. coli*, for example), the *pgk *and *fbaA *genes are cotranscriptionally expressed using two transcriptional promoters, though only one is required to get a strong production of PGK and FBA proteins in the presence of glucose [46]. It has been proposed that when glucose is present in the growth medium, *pts, gapA*, and *pgk *genes are coordinately activated by a mechanism dependent upon the EII^Glc ^protein (coded by the *ptsG *gene) [47]. Our results correlate with these facts, given the increases found in *fbaA, pgk*, and *ptsG *transcript levels.

Surprisingly, only the PGK enzymatic activity was higher (1.54-fold) in CCE14, whereas GAPDH activity was lower (0.64-fold) in this strain. As mentioned previously, it is possible that in the exponential phase, in spite of a higher transcript level of *gapA*, the low GAPDH activity could be related to a redox balance between GAPDH and higher ADH_*Zm *_and PDC_*Zm *_transcripts and enzymatic activities.

Several glycolytic genes showed no significant changes in the transcription level, and some of the transcripts and enzymatic activities did not show the same tendency. This behavior could be related to posttranscriptional regulation and RNA segmentation, leading to the production of individual mRNAs with selective stabilization. These processes allow the adaptation of gene expression to variations in environmental conditions, as has been observed in glycolytic gene expressions in *B. subtilis *[48,49], *Z. mobilis *[50], and *L. delbrueckii *[51]. Another explanation could be that enzyme levels are sufficient to carry out their catalytic role.

On the other hand, only the *pgi *transcript from CCE14 was lower than in the wild type strain. Likewise, the PGI enzymatic activity was slightly lower than that of *E. coli *C. Despite the importance of PGI in glycolysis, little information is available about the regulation of the *pgi *gene. However, due to the results in terms of glucose consumption and ethanol formation rate, the lower transcript and enzymatic activity does not cause any reduction in the glycolytic flux.

#### Entner-Doudoroff and pentose pathways

The *zwf *gene, that codes for glucose 6-P dehydrogenase, plays an important role in the control of carbon distribution at the glucose 6-phosphate node. It directs carbon flux through the oxidative branch of the pentose phosphate pathway (PPP) depending on NADP^+ ^availability. In the CCE14 strain, the transcript level of *zwf *was not different to that of *E. coli *C (Fig. 3); however, ZWF specific enzyme activity was 7-fold higher in CCE14 (Fig. 4). Similarly, transcription of the *gnd *gene, which codes for phosphogluconate dehydrogenase, and one of the isoenzymes that codes for ribose-P isomerase (*rpiB *gene) were higher, 2.01 and 6.31-fold, respectively; while transcript of *rpiA *was lower, 0.71-fold. The isoenzimes transketolases (encoded by *tktA *and *tktB *genes) and transaldolase (encoded by *talA *and *talB *genes) interconnect glycolysis with the oxidative branch of PPP. Our results show that *tktB *and *talA *transcripts were lower, 0.46 and 0.56-fold, respectively, in CCE14; while *tktA *and *talB *were not different when compared to *E. coli *C. These results suggest that the pentose phosphate pathway is very flexible, and it is likely that overall catalytic rates are similar in the two strains tested.

Unexpectedly, genes *edd *that codes for 6-phosphogluconate dehydratase and *eda *which codes for 2-keto-3-deoxy-6-phosphogluconate aldolase in the Entner-Doudoroff pathway were more highly expressed, -2.17 and 2.24-fold, respectively, in CCE14 (Fig. 3). These results suggest that the Entner-Doudoroff pathway is functional, although it has been reported that for aerobic cultures with glucose the carbon flux through this route is not very high [52,53]. In addition, ATP yield is lower through this pathway versus the Embden-Meyerhof pathway [54].

#### Fermentative pathways

In the CCE14 strain, *adhE *(2.09-fold), *fumB *(2.13-fold), *fumC *(1.84-fold), *frdABCD *(2.84, 2.87, 2.38 2.45-fold), and *pflD *(3.43-fold) gene expressions were higher than in the wild type. It is known that *frdABCD *is induced under anaerobic conditions [55], though a recent study showed that this gene can be induced by glucose limitations [56].

It has been demonstrated that the *fumB *transcript is more abundant under anaerobic conditions, and that FNR is necessary as a transcriptional activator [57,58]. In agreement with these reports, the transcription of *fumB *and the anaerobic regulator *fnr *(1.61-fold) were also more highly expressed in the CCE14 strain (Fig. 3). These results indicate that the pathways to produce succinate, formate, and ethanol (for native pathway) are active, although specific enzyme activity values of PFL and LDH and transcript levels of *pflB *and *ldhA *decreased in the exponential phase. It is important to mention that CCE14 produces formate and succinate in low concentrations. These results could indicate a competition phenomenon for pyruvate between PFL and PDC_*Zm*_. However, it is important to consider the disruption of routes to compete for the ethanol production in these conditions such as succinate and lactate pathways. We found that in cultures with high glucose concentrations (100 g/L), the production of succinate increases significantly in strain CCE14 (data no shown). This could be due to osmolarity problems or to a partial limitation of PYK enzyme causing PEP accumulation; hence, the carbon flux may be partially redirected towards succinate formation.

### Metabolic regulation at the enzyme activity level

Enzyme activity levels for CCE14 strain were analyzed in exponential and stationary phases and normalized for values obtained with *E. coli *C (Fig. 4). Enzymatic levels for *pdc*_*Zm *_and *adh*_*Zm *_were normalized with KO11. A *t*-student test with a *p *value of ≤ 0.05 was applied to each set of normalized values to determine statistical differences in enzyme activity levels.

In the CCE14 strain, PTS and PGK enzymatic activities were higher, whereas TPI and PFL activities were lower during the exponential growth phase. These data correlate with the fact that ethanologenic strains consume glucose at a higher rate. In addition, increases in PGK activity suggest that the over expression of the genes coding for PDC_*Zm *_and ADH_*Zm *_modify the ATP/ADP balance. It is known that in aerobic conditions, a high ATP demand causes an increase in glycolytic flux in *E. coli *[59]. For *Lactococcus lactis*, the control of glycolytic flux resides to a large extent in processes outside the pathway, such as ATP consuming reactions and glucose transport [60]. As discussed above, the heterologous ethanologenic pathway increases glycolytic flux with the subsequent increases in ATP production and consumption. Therefore, it appears that cells tend to increase ATP formation through an increase in PGK synthesis; although no increase in PYK activity was found. ATP is also produced when acetate is formed; however, CCE14 does not produce acetate during the exponential phase. A decrease in PFL activity in CCE14 correlates with a strong reduction in formate production (Fig. 2D). On the other hand, the observed 50% reduction in the TPI specific enzyme activity does not reduce glycolytic flux. A large increase in ZWF specific activity was found, and was discussed above.

A comparison of enzymatic activity data between CCE14 and *E coli *C during the stationary phase, indicates higher values of PTS, FDP, GAPDH, PGK, and PYK for the ethanologenic strain. The LDH enzymatic activity was lower in CCE14. It is known that lactate dehydrogenase is allosterically activated by pyruvate [61]. Lactate production was found only during the stationary phase of two cultures. Pyruvate formation in *E. coli *C correlates with lactate production (Fig. 2C–G), and lower levels of this metabolite in CCE14 correlate with a 40% lower LDH specific activity (Fig. 4). These results suggest that a higher ethanol formation rate, *i.e.*, higher PDC_*Zm *_and ADH_*Zm *_specific activities originate higher rates of glucose transport, ATP synthesis, and NAD-NADH+H turnover.

## Conclusion

A higher glycolytic flux in CCE14 results from increased chromosomal expression of *pdc*_*Zm *_and *adh*_*Zm *_genes and higher specific enzyme activities of heterologous PDC_*Zm *_and ADH_*Zm *_enzymes involved in ethanol formation. These results indicate that under the conditions used in this study, the glycolytic flux is controlled by reactions outside this pathway, that is, by the fermentative heterologous route. The metabolic adjustments carried out in the cell entail low organic acid production and an increase in the ethanol formation rate, as well as higher ethanol yield (90% of the theoretical) in glucose-mineral media when compared with previous engineered efficient strains, such as KO11 (70% of the theoretical yield). In spite of the higher PDC_*Zm *_and ADH_*Zm *_transcript and enzymatic activities in the CCE14 strain, the differences are mediated by higher glucose transport rates and an increase in the turnover rate of NAD-NADH+H^+ ^and ATP.

Overall, these results also show that *E. coli *glycolytic enzymatic activities under fermentative conditions are sufficient to contend with increases in the rates of glucose consumption and higher transcript and enzymatic activities of the heterologous ethanol pathway. Also, the study provides the basis for the implementation of appropriate genetic modifications to increase the ethanol yield when mineral media is used; for instance, the disruption of succinate and lactate pathways that compete for ethanol production.

## Methods

### Bacterial strains, media and growth conditions

*E. coli *strains used in this work are listed in Table 1. With the purpose to have an ethanologenic strain with different PDC_*Zm *_and ADH_*Zm *_enzymatic levels, a new strain CCE14 was constructed integrating into the chromosome the pyruvate decarboxylase (*pdc*) and the alcohol dehydrogenase (*adh*) genes of *Zymomonas mobilis *under the control of the *pfl *native promoter. This chromosome integration was made with pLOI510 as described previously, [3]. The vector pLOI510, was constructed to allow direct selection for the integration of *pdc *and *adhB *of *Z. mobilis *genes into the *pfl *region of the chromosome by using a DNA fragment which lacks a replicon [3]. Transformants were screened for chloramphenicol resistance (20 μg/ml) and CO_2 _production in tubes, and subsequently tested for ethanol production in mini-fermentors with 20 g/l of glucose.

**Table 1:** *Escherichia coli *strains used in this study

Strain	Relevant features	Reference
*E. coli *C	Wild type	ATCC 8739
CCE14	*E. coli *C: *pflB::pdc adhB cat*	This work
KO11	*E coli *W: *pflB::pdc adhB cat, Δfrd*	Ohta *et al *1991 Jarboe *et al *2007

All stock cultures were stored at -70°C in Luria Broth (LB) medium [62] containing 40% glycerol. To develop inocula, cells were transferred twice on LB-agar plates supplemented with 20 g/L of glucose-chloramphenicol (20 μg/ml), and no chloramphenicol for *E. coli *C. Single colonies were transferred to overnight cultures in shake flasks (35°C, 120 rpm), containing glucose (20 g/L) in mineral M9-medium [62]. M9-medium contains: 6 g/L Na_2_HPO_4_, 3 g/L KH_2_PO_4_, 1 g/L NH_4_Cl, 0.5 g/L NaCl. The following components were sterilized by filtration, and then added (per liter of final medium): 2 ml of 1 M MgSO_4_7H_2_O, 1 mL of 0.1 M of CaCl_2_, 1 mL of 1 mg/mL thiamine-HCl. These cells were harvested in exponential growth phase by centrifugation and used to inoculate non-aerated mini-fermentors [63], containing 200 ml of M9-medium with 40 g/L of glucose. Starting OD_600 _was 0.1, chloramphenicol (0, 40 and 20 μg/ml) was included for strains C, KO11 and CCE14, (respectively), cells were cultivated at 35°C, 100 rpm, and the pH was maintained at 7 by the automatic addition of 2 N KOH. All cultures were carried out in triplicate.

### Analytical methods

Samples were periodically taken from cultures to measure optical density at 600 nm using a spectrophotometer (Beckman DU-70, Palo Alto, CA) and the dry cell weight was calculated using a previously determined conversion factor of 1 OD_600 _= 0.37 g/L. An HPLC system (600E quaternary bomb, 717 automatic injector, 2410 refractive index, and 996 photodiode array detectors, Waters, Milford, MA) and an Aminex HPX-87H column (300 × 7.8 mm; 9 μm) (Bio-Rad, Hercules, CA) were used to separate and quantify D-glucose, formate, acetate, succinate, and lactate concentrations. Running conditions were: mobile phase, 5 mM H_2_SO_4_, flow 0.5 ml/min, and temperature 50°C. Under these conditions glucose was detected by refractive index, and organic acids were identified by photodiode array at 210 nm. Ethanol was quantified by gas chromatography (Agilent 6850, Wilmington, D.E.), using 1-butanol as internal standard.

### Preparation of cell extracts and enzymatic assays

Samples were taken at the mid-exponential and stationary phases. All operations were carried out at 4°C. 1 mL of cell culture was harvested by centrifugation at 10,000 × g for 10 min, washed twice with 1 mL of 100 mM Tris-HCl (pH 7.0) containing 20 mM KCl, 5 mM MnSO_4_, 2 mM DTT and 0.1 mM EDTA, and then suspended in 1 mL of the same buffer. Cells were disrupted by four sonication steps (15 s each) in an ultrasonic disrupter (Soniprep 150, UK). The cell debris was removed by centrifugation; 10 min at 10,000 × g. The resulting crude extracts were used immediately for determination of enzymatic activities and protein, or stored at -20°C.

Enzyme activities were measured spectrophotometrically at 340 nm in a thermostatically controlled (30°C) spectrophotometer (BioMate 5, ThermoSpectronic, NY). All compounds of the reaction mixtures were pipetted into 1 cm light path cuvettes, reactions were initiated by adding the cell extract or substrate to give a final volume of 1 mL. The millimolar extinction coefficient for NAD^+^, NADH, NADP^+ ^and NADPH is 6.22 cm^-1^. mM^-1^.

The assay conditions for glucose:PEP phosphotransferase (PTS), 6-phosphofructosekinase (PFK), fructose-1,6-bisphosphatase (FDP), glucose phosphate isomerase (PGI), fructose bisphosphate aldolase (FDP aldolase), glyceldehyde-3-phosphate dehydrogenase (GAPDH), triose phosphate isomerase (TPI), 3-phosphoglycerate kinase (PGK), pyruvate kynase (PYK), 6-phosphogluconate dehydrogenase (ZWF), pyruvate-formate lyase (PFL), and lactate dehydrogenase (LDH) were measured based on the methods used by Peng *et al *[64]. The assay conditions used for alcohol dehydrogenase (ADH_*Zm*_) and pyruvate decarboxylase (PDC_*Zm*_) were based on the methods reported by Conway *et al *[65]. Phosphoglycerate mutase was measured based on the method of Maitra *et al *[66]. Protein concentration was estimated by the Bradford method, [67], with bovine serum albumin used as the standard. Each assay was performed three times for the same culture, from three independent experiments. A *t*-student test with a *p *value of ≤ 0.05 was applied to each set of normalized values in order to determinate statistical differences in enzyme activity levels.

### RNA extraction and cDNA synthesis

Total RNA extraction was performed using hot-phenol equilibrated with water, precipitated with 3 M sodium acetate and ethanol, and treated with DNase kit (DNA-free™, Ambion; [33]. RNA integrity was tested by densitometry in 1.2% agarose gels. RNA quantification was performed by absorbance at 260/280 nm. cDNA was synthesized using RevertAid™ H First Strand cDNA Synthesis kit (Fermentas Inc.) and a mixture of specific DNA primers. The sequences of the primers used for cDNA synthesis were those reported by Flores *et al *[33], except for *pdc*_*Zm *_(5'-GACAAAGTTGCCGTCCTCGT and 5'-ATGGTAGCAACTGCGCCAC) and *adh*_*Zm *_(5'-TTACCCCGATGGTTTCCGT and 5'-TTCAAATGCGTGGGTCAGAG) genes. cDNA obtained in this way was used as template for RT-PCR assays. Reproducibility of this procedure was determined by performing two separate cDNA synthesis experiments from the RNA extracted for each strain. Similar results were obtained for the transcription of all genes that were measured.

### Real-time PCR

Real-time PCR (RT-PCR) was performed with the ABI Prism 7000 Sequence Detection System (Perkin Elmer/Applied Biosystems, Foster City, CA), using the SYBR Green PCR Master Mix (Perkin Elmer/Applied Biosystems, Foster City, CA) and amplification conditions described by Flores *et al *[33]. The primers for specific amplification were designated using the Primer Express software (Perkin Elmer/Applied Biosystems, Foster City, CA). The size of all amplimers was 101 bp. The final primer concentration, in a total volume of 15 μl, was 0.2 μM. Five nanograms of target cDNA for each gene was added to the reaction mixture. All experiments were performed in triplicate for each gene of each strain, obtaining very similar values. A non-template control reaction mixture was included for each gene. The quantification technique used to analyze data was the $2^{-\Delta \Delta {\text{C}}_{\text{T}}}$ method described by Livak and Shmittgen [68]. The data were normalized using the *ihfB *gene as an internal control (housekeeping gene). We detected the same expression level of this gene in all the strains in the conditions in which the bacteria were grown. For each analyzed gene in all strains, the transcription level of the wild type gene, considered as one, was used as the control to normalize the data. Data is reported as relative expression levels compared to the expression levels of *E. coli *C. A *t*-student test with a *p *value of ≤ 0.05 was applied to each set of normalized values in order to determinate statistical differences in expression levels.

## Competing interests

The authors declare that they have no competing interests.

## Authors' contributions

MOT Constructed the ethanologenic strain, carried out the kinetic, RT-PCR and enzyme characterization, the data analysis and drafted the manuscript. NF Participated in RT-PCR analysis and proofreading the manuscript. AE Participated in RT-PCR technique and proofreading the manuscript. GHC Participated in HPLC analysis. FB Reviewed and commented the manuscript. GG Reviewed and commented the manuscript. AM Conceived the study, designed and supervised the experiments, participated in results analysis and helped with manuscript preparation.
